# What Does it Take to Become a Team? Group Work in a Final Year Food Science Capstone Module

**DOI:** 10.1111/1750-3841.70688

**Published:** 2025-11-23

**Authors:** Maricel Krügel, Charmaine van der Merwe, Hanelie Adendorff

**Affiliations:** ^1^ Department of Food Science, Faculty of AgriSciences Stellenbosch University Stellenbosch South Africa; ^2^ Centre for Teaching and Learning Stellenbosch University Stellenbosch South Africa

## Abstract

Trial design and new product development (NPD) is a final‐year Food Science module where students are assigned to groups for the year to develop new food products. Group work is an outcome of the module. Group work skills development is often overlooked during curriculum design in favor of disciplinary outcomes. This raises the question: how do we best facilitate student learning toward these outcomes? In this study, group work is designed to include learning opportunities to help students to become effective teams working towards a common goal. Two analytical procedures were used to look at the incorporation and integration of group work: (1) thematic analysis of student reflections using the Johnson and Johnson Cooperative Learning framework; and (2) analysis of the NPD curriculum, using the knowledge‐action‐self framework as a lens. A key finding is that where teamwork is deliberately taught and included as an outcome, a skilled facilitator is needed to help students make sense of situations that they cannot resolve themselves. Furthermore, regular opportunities for reflection, which can help them make sense of their own learning and growth, need to be created. It takes a collective act for the individual to learn to become a team player.

**Practical Applications:**

This research can help universities and educators better prepare Food Science students for real‐world careers by teaching them how to work effectively in teams. By integrating teamwork, reflection, and facilitation into the curriculum, students gain valuable interpersonal and problem‐solving skills that are essential in modern food industry roles.

## 1 Introduction

Food Scientists need many diverse skills to meet the needs of a globalized food and drink sector, from food‐specific, scientific skills (Ellis 2024) to the more intuitive soft skills (Flynn et al. 2017), such as group work, communication, and leadership (Bohlscheid and Clark 2012). Increasingly, employers are demanding skills from graduates that are outside the subject area of their course of study in higher education, with increasing importance being placed on generic skills. Key examples in this regard are group work and communication skills. It should come as no surprise that team members who can work cohesively together have been found to be more successful in the product development process (Stewart‐Knox and Mitchell 2003; Vogler et al. 2018). Working in a team enables individuals to collectively use their uniqueness, varied skills, expertise, and knowledge to solve complex problems from different perspectives (Gallup n.d.). It is argued that the “knowledge‐ and technologically driven” nature of the current century implies a need for higher education teaching and learning programs to develop “core subject knowledge as well as new media literacies, critical and systems thinking, interpersonal, and self‐directional skills” (UNESCO n.d.). Arguing that group work is a key “method that supports the learning of such skills,” UNESCO (n.d.) thus calls for greater use of inquiry‐based, collaborative group projects, focusing on ‘real‐world issues and questions’ to address the needs of the 21st century.

The Institute of Food Technologists (IFT) (Bohlscheid and Clark 2012), Weston et al. (2019), and Stellenbosch University, in their Strategy for Teaching and Learning (Stellenbosch University 2017), have also identified group work as a key component to be part of professional competence. Group work is thus included as a key outcome of the module trial design and NPD. Although McSweeney et al. (2017) found that capstone experiences like the NPD module (1) allow students to integrate and apply knowledge gained throughout the course and (2) combine graduate capabilities and employability skills. Modipane (2020), however, warns that group work activities often tend to favor disciplinary outcomes over group work skills. The NPD experience over the years has also shown that students cannot necessarily work together effectively in groups, especially in fast‐paced, dense, and highly technical subjects. Furthermore, if the group work element of the process fails, it was observed in the NPD module that the disciplinary outcomes often also suffered. Group work was thus purposefully and explicitly included in the NPD curriculum, with all groups working toward a common goal (Johnson and Johnson 2009).

Trial Design and New Product Development is a final year undergraduate Food Science module that forms part of the BSc Food Science 4‐year degree program. This project‐based module allows students to gain insight into the entire food product research and development process. Students in NPD are assigned to groups that remain the same throughout the year and must integrate all the fundamental food science principles in order to research and develop a new food product to simulate the real‐life workspace.

At the start of the NPD module, students are divided into groups in which they must work together for the entire year. To ease them into this, students are introduced to the concept of personal communication styles (Alessandra n.d.) and how this could impact group work. Each of the four communication styles, namely, relator/supporter, socializer/promoter, thinker/analyzer, and director is introduced, defined and discussed. Students are then given time to reflect on their own style before working in their groups to map out the communication styles represented within their team. Using the unique group profile, students identify and discuss potential areas of conflict that could emerge from their unique combination of personal communication styles (an example is presented in the results and discussion section). This is followed by looking at different elements or aspects of teamwork, such as conflict management and decision making using the six thinking hats of Edward De Bono as an example (De Bono 1985). Conflict scenarios that manifested in previous years of teaching the module are used to demonstrate the principles of conflict management and to aid the learning process. Groups are advised to assign group roles and to sign a group contract. They are also alerted to the typical stages of group formation (Tuckman and Jensen 1977), i.e., the forming, storming, norming, and performing stages. Lastly, it is recommended that groups carefully consider the communication channels and online platforms they plan to use.

Food Science specific and technical aspects of NPD, such as the brief, concept development, target market, formulation, costing, processing, microbial safety, regulations, typical nutritional information on the label, sensory analysis and packaging are only addressed after addressing the group work specific content mentioned above. Throughout this process, there are multiple times when the students must submit individual reflections on how they and their group are doing. The actual product development process is continuous, spanning a whole academic year. Throughout this entire process, weekly or bi‐weekly meetings were scheduled, involving the course facilitator and each group. During these brainstorming sessions, weekly achievements and problems were highlighted and discussed. This included group‐related and more technical, NPD‐related, challenges. The facilitation was thus purposefully designed to include learning opportunities that focus on the skills needed to perform in groups (Johnson et al. 2007). Although these group discussions contributed to the overall functioning of the groups, the discussions were not recorded and not included as data for this study.

The purpose of this study was to gain a better understanding of how group work can be effectively incorporated into an applied Food Science curriculum. To this end, we (1) set out to form a better understanding of how the experience of students in this study related to Johnson et al's (Johnson et al. 2007) requirements for effective group work; and (2) we used the Barnett et al. (2001) model of curricular change to determine the extent to which the NPD curriculum structure supports group work as an outcome. These two lenses were therefore used to reflect on our overarching research question: How can group work be incorporated and integrated into a Food Science curriculum? What do students value in terms of teamwork? Here, the focus was on what made students feel that they were part of a team or not. What are the dominant domains, as mentioned by Barnett et al. (2001), in our curriculum? Here, the focus was, among others, on curriculum progression over the course of the four‐year study programme.

## 2 Materials and Methods

The methodology followed a two‐pronged approach: looking at the elements of effective group work through the lens of Johnson et al. (2007) and looking at the nature of the NPD curriculum using Barnett et al's. (Barnett et al. 2001) model.

### 2.1 Effective Group Work

Cooperative and collaborative learning are often used interchangeably to describe group work or learning in groups. Even though much has been written about both concepts, there still seems to be a lack of definitional clarity (Laal and Laal 2012). Bruffee (1995), for example, holds that there is little difference between cooperative and collaborative learning, other than the assumptions about knowledge made by the two groups of practitioners. Jacobs (2015) similarly argues for focusing on the implementation of group work approaches rather than on ‘the label educators use’ to describe the approach. Hence, even though elements of both approaches were present in the design of the NPD module, in this paper we use the term cooperative learning, in line with the work of Johnson et al. (2007). By their definition, cooperative learning is aimed at fostering interdependence. Key features include the use of assigned group roles (Davidson and Major 2014) and working toward a shared learning outcome. It requires commitment and accountability to one another, as well as students working together to maximize their own and each other's learning (Johnson et al. 2007).

Johnson and Johnson (1999) use social interdependence theory to unpack five basic elements needed to understand the dynamics of cooperative learning. Social interdependence exists when the accomplishment of each group member's goals is affected by the actions of the other members. At the heart of cooperative effort is *positive interdependence*. Positive interdependence exists when individuals perceive that they can reach their goals only when the other group members also reach their goals. The second element is *individual accountability*. This exists when the performance of individual group members is assessed, but the results are shared with the group, holding everyone responsible for contributing to the success of the group. The third basic element is *promotive interaction*. This may be defined as individuals encouraging and facilitating each other's efforts to complete tasks in order to reach the group's goals. The fourth element is the *appropriate use of social skills*, such as leadership, decision making, trust‐building, communication, and conflict management skills. These skills must be taught just as purposefully and precisely as academic skills. The last element is group processing, where effective group work is influenced by whether the groups periodically reflect on how well they are functioning and how they may improve their learning processes (Johnson and Johnson 1999).

### 2.2 The Nature of the NPD Curriculum

**FIGURE 1:**
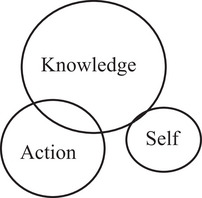
Curriculum: science and technology schema (Barnett et al. 2001).

Barnett et al. (2001) propose that curricula consist of three interacting domains namely: knowledge, action, and self. The knowledge domain represents the discipline‐specific competencies required to develop subject specialists. The action component represents the competencies that are acquired through the act of doing something. Lastly, the self‐domain is where the educational identity, in relation to the subject area, is developed. The weight of these domains varies across curricula and can therefore either be separated or integrated. According to Barnett et al. (2001), science and technology curricula are typically heavily weighted toward the knowledge domain, with little or no integration between the knowledge, action, and self domains (Figure 1).

### 2.3 Methods

For the research question, the study followed a phenomenological approach involving an analysis of the consciously lived experiences of the students within a qualitative paradigm (Daniel and Harland 2018). Here, the focus was on how the students conceptualized what it means to be part of an effective team.

During their individual reflections throughout the NPD year, students were asked to respond to the following open‐ended questions:

1. How effectively does your group work?
2. Are there any behaviors of any of your group members particularly valuable or detrimental to the team?
3. What did you learn from working in your team?
4. How do you see yourself as a team member?

**FIGURE 2:**
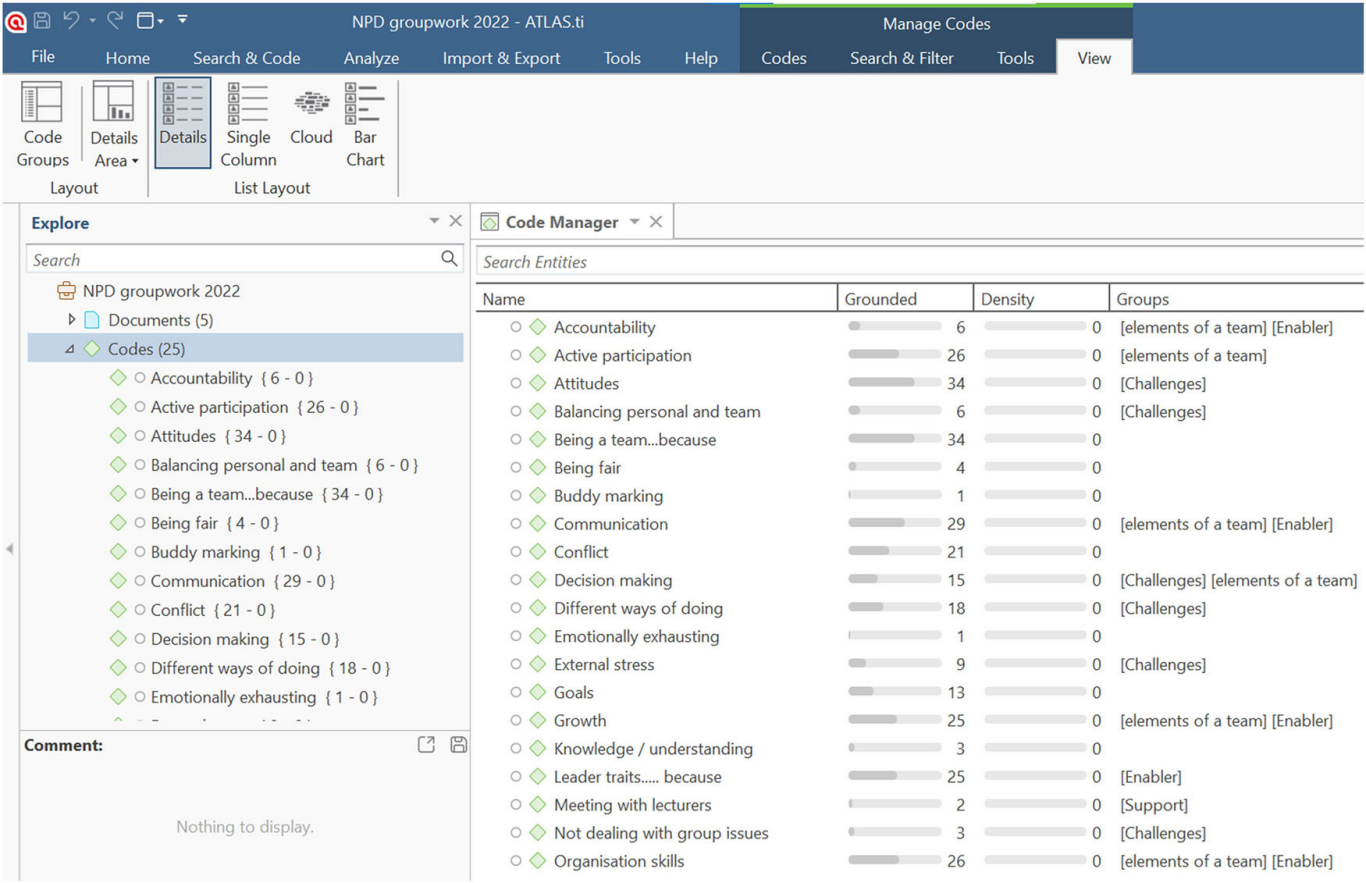
A screenshot of the coding of the reflections using the software, ATLAS.ti.

After obtaining ethical and institutional permission, fifty of the fifty‐two NPD students agreed to the use of their individual reflections as a data source for this study. No permission was obtained to record data from the weekly team meetings. The research commenced with a thematic analysis (Braun and Clarke 2006) of the reflection data, followed by coding using ATLAS.ti 9 (Figure 2). To minimize bias in the analysis, the three researchers independently reviewed the student reflections and generated preliminary themes without consultation. These themes were then compared in a joint discussion to identify areas of agreement and divergence. Through an iterative process of consensus‐building, a shared coding framework was developed and inductively applied to the reflections. Coding decisions were documented, and any disagreements were resolved collaboratively to ensure transparency and rigor in the analysis.

#### 2.3.1 Subjects

This study was reviewed and approved by the Research Ethics Committee: Social, Behavioral and Education Research of Stellenbosch University, approval number TL‐2019‐9195. Informed consent was obtained from each subject prior to their participation in the study.

## 3 Results and Discussion

### 3.1 Results

#### 3.1.1 Using the Johnson and Johnson Cooperative Learning Framework as a Lens for Effective Group Work

**FIGURE 3:**
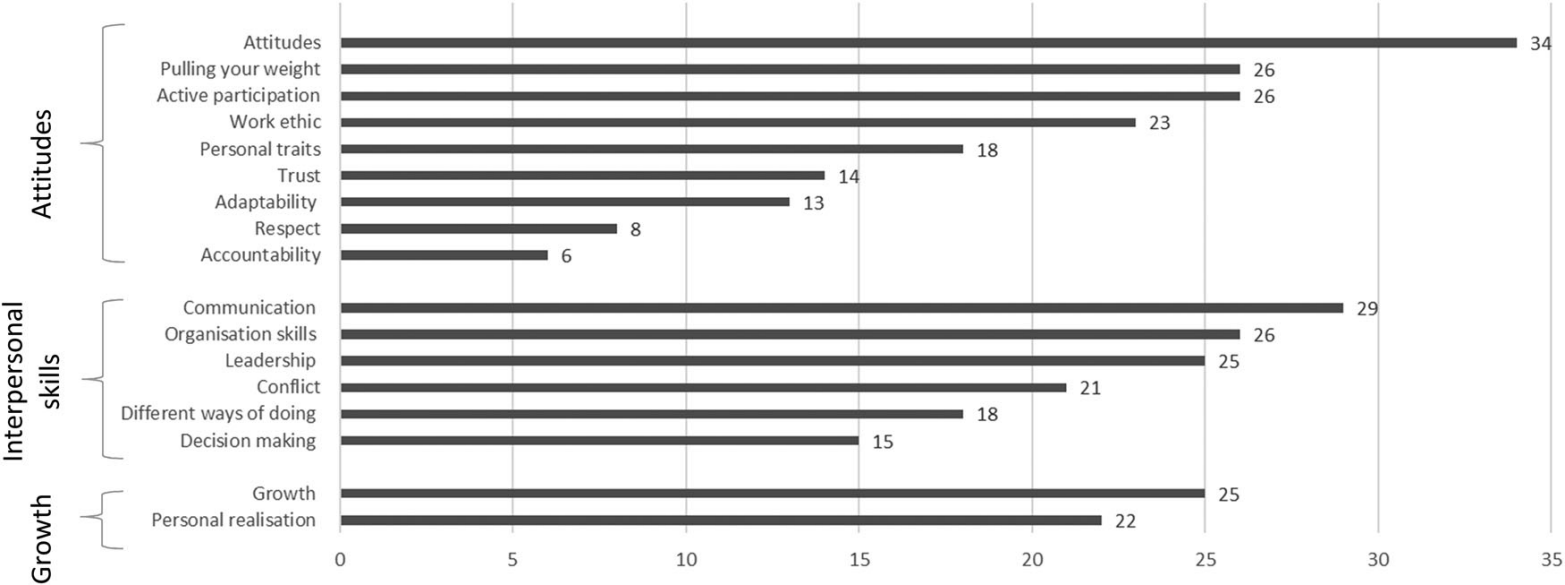
Frequency of codes related to what make groups feel like a team.

Student comments about what makes them feel like a team included references to the importance of active participation, positive attitudes, effective communication, growth, leadership, organization, personal realization, and the absence of conflict (Figure 3).

These comments seem to center around three broad themes central to students experiencing group work as teamwork (Figure 3): (1) attitudes and characteristics, with specific references to pulling your weight, active participation, work ethic, personal traits, trust, adaptability, respect and accountability; (2) interpersonal skills, such as effective communication, organization skills, leadership, conflict management, different ways of doing, and decision making; and (3) growth and self‐realization or personal growth, which seems to refer to them changing as a result of their participation in the team. When applying the Johnson et al. (2007) framework for effective cooperation as a lens, all five aspects of the framework become visible in the student feedback. References to active participation, work ethic, and pulling your weight were coded as examples of positive interdependence. This is in line with the suggestion of Johnson et al. (2007) that positive interdependence exists when each student plays a unique and complimentary role with effective division of labor. This is exemplified in the following quote about task‐interdependence:

Yes, if someone is not doing their part, then sometimes you cannot continue your work or put more workload on other team members.

Team members also internalized the idea that each member contributed, either positively or negatively, to the quality of their final product, an example of outcome interdependence. In one quote a specific team member's “behavior towards” their assigned task was described as “apathetic” and “lowering the standard of [their] progress report”.

Beyond these references to outcome and task‐interdependence (Johnson et al. 2007), the following quote also mentions role‐interdependence, highlighting the unique role or contribution, in terms of overall teamwork, of each team member:

- “Is a good leader who maintains a positive outlook and keeps everyone in the group positive. She handles situations diplomatically to keep all group members happy.”
- “Always helps out where she can; she is well prepared and knowledgeable on many subjects.”
- “Is good at admin. She has set many documents in place that improved our group's effectiveness and helps to keep track of the work done.”
- “Is knowledgeable on many subjects; he can always find solutions to problems and maintains a positive attitude.”

In general, student responses in this area centered around helping “each other to finish tasks effectively” and “maintain[ing] a high quality,” which is well aligned with the idea that they ‘sink or swim together’ (Johnson et al. 2007).

The second element of effective group work, individual accountability, addresses the idea of group members holding one another responsible or accountable for the individual tasks assigned to them. It implies that group members develop the ability to identify individual needs for “assistance, support, and encouragement” (Johnson et al. 2007) required to complete the project. The students in this study mentioned being aware of one another's strengths and weaknesses and using that as a basis for assigning tasks, “to make each group member a stronger individual in his or her right” (Johnson et al. 2007). Student quotes in this category mentioned learning how to use their “strengths and weaknesses when working in a group” and that “everyone has their own unique qualities”.

References to collectively understood goals, as well as support from their team members, team coordinators, and the lecturer were coded as examples of promotive interaction, the third element of effective group work. This element is defined as the ability to “encourage and facilitate each other's efforts to complete tasks and achieve the group's goals” (Johnson et al. 2007). This was exemplified in quotes such as:

“I have learnt a few things during this project – one is that one can indeed depend on another member to help you achieve a certain goal. I usually take the majority of all of the work on myself in any group project and work hard without asking for help, but now I have learnt that everyone wants to help achieve the same goal.”

Another is that “everyone has unique skills that are all essential in order to have a successful project.” Most of the references in the data clustered around the fourth element, social skills. This is also the area that most of the teamwork preparation and structured input in the module focuses on. Codes in this category included organizational skills, trust, leadership, and communication. Students, amongst others, referred to learning to “listen to each other and respect[ing] everyone's ideas” as well as learning to “rely on one another and trust[ing] one another to deliver work of adequate quality.”

The fifth element of effective teamwork, group processing, offered an interesting perspective on the student experience and development. Two key themes that manifested in this category are self‐realization and growth, with self‐realization referring to learning to see themselves as active and effective team members, and growth describing, amongst others, learning how to use criticism, and not take it too personally. References in this area often represented a change in identity for students, i.e., learning to be patient and to stay calm, learning to listen to one another, as well as learning to be more confident and to “express concerns in a constructive way.” They also had to overcome fears about their ability to contribute, i.e. “not hav[ing] self‐doubt when I have something to say because it is valuable.” One learned that they “cannot always control everything, especially when in a team,” and that “some things require a group of people to have a better outcome**.”** Another student realized that other students found him/her very intimidating. They also, importantly, highlighted the importance of “properly address[ing] issues as soon as they pop up.” Our data clearly indicate that the student experience in the NPD module is aligned with Johnson et al's. (2007) five requirements for effective cooperation.

Johnson et al. (2007) mention the need for groups to “periodically reflect on how well they are functioning.” Modipane (2020) and Cochran‐Smith and Lytle (1999) also highlight the value of regular reflection for improving group work practices. The individual student reflections in this study offered a tool for the facilitator to get insight into the process of group formation and individual growth. These reflection opportunities allowed students to also consider their own growth and contribution to the team. One student, for example, reflected that “teamwork is really difficult, as not everyone in the team wants to achieve the same level of achievement in the project,” but that they “have learned to enjoy working with people who [they] thought [they] would be arguing with a lot.” This supports the idea that regular reflection can help students make sense of the group dynamics and requirements for group work success.

**FIGURE 4:**
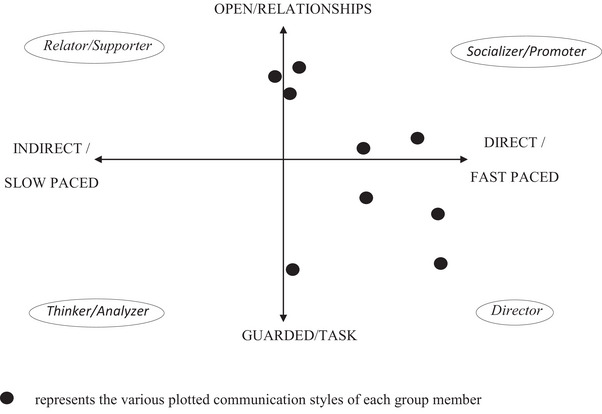
An example of a plotted communication style of one of the NPD groups.

An unexpected but important finding is the importance of the tutoring role of the facilitator. Students, for example, mention the key role played by the lecturer. i.e., “We are working much more effectively after the meeting with [our lecturers],” and “after the meeting with [our lecturers] our group work improved alot.” A tutor in the higher education setting is described as a ‘subject matter expert, resource guide, and facilitator of learning in the group’ (O'Neill et al. 2005). Group work tutors, in this context, are facilitators of the process of learning to function effectively in a team, thus “promot[ing] group processing” rather than imparting subject knowledge. Learning group work thus requires a tutor with an understanding of how and where groups can get stuck during the process of learning to work in a group. For example, during one of the weekly brainstorming sessions with one of the groups, it became clear that they were struggling with internal conflict. A plot of their communication styles (Figure 4) revealed that they all had very similar, fast‐paced and direct, communication styles.

A typical assumption could have been that the group's problems arose because there were too many strongly opinionated leaders in the group, each wanting things their way. But, in conversation, the facilitator learned that the opposite was true. Fearing that they would come across as too strongly opinionated and direct, many of the group members overcompensated by not sharing their ideas and opinions. This negatively impacted active decision making and communication, with a resultant increase in frustration levels, especially when crucial decisions had to be made. The value of a tutor, the facilitator in this case, was crucial in helping this group identify and address their problems. This example serves as a reminder of the need for tutoring when teaching group work skills. More than one group referred to how they worked much better after meeting with the lecturer in a similar way (see quotes above).

#### 3.1.2 Using the Knowledge‐Action‐Self Framework as a Lens for Curriculum Design

Applying the curriculum model of Barnett et al. (2001) to the Food Science programme, shows that the first three years of the curriculum mostly follow the authors’ depiction of the typical Science, Technology, Engineering and Mathematics (STEM) curriculum. The curriculum for these three years is weighted towards knowledge, specifically knowledge of basic sciences and knowledge of food science, with some action, manifested in the requirement that students apply the content knowledges to given situations or problems. Although group work is often used, it is seldom an outcome. Thus, self‐domain is far less prominent with identity building mostly taking place coincidentally, rather than being a planned part of the curriculum. Furthermore, these domains are mostly kept separate in the first three years.

**FIGURE 5:**
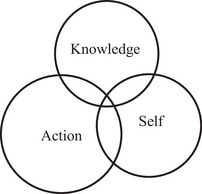
Schematic representation of the NPD curriculum, which is similar to the professional schema depicted by Barnett et al. (2001).

However, with the NPD capstone project in the fourth year, the curriculum shows a substantial shift, with the action and self domains growing in prominence and being planned into the curriculum. Figure 5 illustrates the NPD curriculum domains, which correspond with how Barnett et al. (Barnett et al. 2001) depict professional subject areas. The knowledge domain remains important, and now includes knowledge of working in a team, such as communication and organizational skills, but it is no longer the most important domain. In the NPD project, the action domain is the driving force, since students must produce something as a team. The self‐domain is also structured into the process through regular student reflections and a focus on topics such as their personal communication and conflict management styles. The fourth‐year curriculum also includes far greater integration of the three domains than in the previous three years of the curriculum.

### 3.2 Discussion

In the first three years of the Food Science programme, the focus is mostly on individual learning. Even though many of the modules might include occasional and informal group work and teamwork activities, these are not key requirements for success. Students could pass by just focusing on themselves and on the knowledge that they must acquire. However, with the capstone project in the fourth year, there is no bypassing the collective. Here, individual success entirely depends on the success of the team. Furthermore, with action, rather than knowledge, being the focus in the fourth year, success requires them to integrate and internalize theoretical and practical aspects of both content knowledge and group work knowledge. It needs to be noted that a key outcome, and the purpose of the shift in the curriculum, is teamwork. The NPD project creates opportunities for students to achieve this through practicing and internalizing the key elements of working in teams and integrating that into themselves as individuals, through the curricular shift in the final year. Using Barnett's et al. (2001) model suggests that effective integration of group work into a curriculum will require a greater focus on action and self. The Food Science programme progresses from an individual focus, through the collective in the fourth year, to students accepting the teamwork outcome as individuals. The data in this study shows that the use of reflection enabled students to recognize areas for growth in terms of teamwork, both for themselves and for their team. This is evident in the frequency of codes, in their reflections, related to growth and personal realization (Figure 3).

The positive mention of the role of the lecturer in the student reflections could possibly be understood in the context of this shift to more teamwork related action. In disciplinary teaching contexts, there would usually be mentors or tutors who could help students get out of stuck places, specifically while learning to apply concepts. Similarly, when group work is the concept that is applied, this study shows that students would stand to gain from a facilitator who could help them get out of stuck places (Fung and Lui 2016), such as handling conflict situations. Take the example shared earlier in this paper (Figure 4): the group that had many members with director communication styles. It was only in the facilitator's interaction with the group that they were able to figure out what was going on and why they were struggling.

Our original question was how to incorporate group work in a Food Science or applied science curriculum. Our data highlighted all the elements in the Johnson et al. (2007) model, confirming their importance in creating effective spaces for learning group work. Using the Barnett et al. (2001) model, it can be concluded that effectively integrating group work into a curriculum, would imply a stronger emphasis on both the action and self domains. In this study, this was achieved through group work related tasks, enabled by a skilled facilitator, and regular, guided reflection to help students process their experiences and integrate what they have learned.

At all levels of curriculum formation (course team, department, institution, national policy) curricula should be understood as embracing the three domains of knowledge, action and self. The challenge in developing curricula is not just to ensure that these three domains are adequately represented in the curriculum but that the moments of these three domains are, in due measure, integrated (Barnett et al. 2001). To ensure that graduates can compete and succeed in the modern world of work, it is necessary for them to possess a range of professional competencies and not only the traditional technical skills which are mostly driven by knowledge. Yonai et al. (2024) found that by including authentic learning, namely soft skills and hard skills, affected the self‐efficacy and career aspirations of science students. Curricula need to respond in a world of change where students will be overwhelmed by more facts, data, evidence tasks, and arguments (Barnett 2000). They must be able to navigate and orient themselves as well‐rounded graduates and that can be achieved more easily if curricula encompass and integrate the knowledge, action, and self domains. In Food Science, you will always be part of a group, either in quality as part of a Food Safety Management Group or perhaps in NPD. You will never work in a silo; therefore, the sooner students get a good understanding and experience of how to work as part of a group to become a team, the better for themselves as well as the employer.

## 4 Conclusion

We can teach students about group work skills, but if we really want them to internalize this and integrate it into their identity, we need to allow them to experience long term, high stakes group work (Lotrecchiano et al. 2023). In such groups, they cannot bypass the collective or the typical stages of group formation (Tuckman and Jensen 1977) but have to work through the conflict of the storming stage to finding norms for their team. To this end, we conclude that curricula will need to shift to a greater focus on the self and action domains whilst paying attention to the elements of effective collaboration Johnson et al. (2007). Furthermore, where group work is deliberately taught and included as an outcome of a study programme, our data suggests the need for a skilled facilitator to help students make sense of situations that they cannot resolve themselves, as well as regular opportunities for reflection, which can help them make sense of their own learning and growth. Johnsen et al. (2024) also found that students who participated in project‐based group work showed significant improvement in interpersonal and conflict management, and that higher education teachers of facilitators should be reminded to guide and support both their students’ learning and group processes.

It takes a collective act for the individual to learn to become a team player. The success of this collective act rests on intentionally redesigning the curriculum and including tutoring by a skilled facilitator as well as regular opportunities for guided reflection.

## Author Contributions

**Maricel Krügel**: conceptualization, investigation, funding acquisition, writing – original draft, methodology, validation, visualization, writing – review and editing, software, formal analysis, project administration, data curation, supervision, resources. **Charmaine van der Merwe**: conceptualization, investigation, writing – original draft, methodology, validation, visualization, writing – review and editing, software, formal analysis, resources, data curation. **Hanelie Adendorff**: methodology, software, writing – review and editing, visualization, validation, data curation, resources, formal analysis, writing – original draft, investigation.

## Conflicts of Interest

The authors declare no conflicts of interest.
